# Storage conditions determine the characteristics of red blood cell derived extracellular vesicles

**DOI:** 10.1038/s41598-022-04915-7

**Published:** 2022-01-19

**Authors:** Tímea Bebesi, Diána Kitka, Anikó Gaál, Imola Csilla Szigyártó, Róbert Deák, Tamás Beke-Somfai, Kitti Koprivanacz, Tünde Juhász, Attila Bóta, Zoltán Varga, Judith Mihály

**Affiliations:** 1 Institute of Materials and Environmental Chemistry, Research Centre for Natural Sciences (RCNS), Magyar tudósok körútja 2, 1117 Budapest, Hungary; 2 Hevesy György PhD School of Chemistry, ELTE Eötvös Loránd University, Pázmány Péter sétány 1/A, 1117 Budapest, Hungary; 3 Institute of Enzymology, Research Centre for Natural Sciences (RCNS), Magyar tudósok körútja 2, 1117 Budapest, Hungary

**Keywords:** Biophysics, Chemical biology, Materials science, Nanoscience and technology

## Abstract

Extracellular vesicles (EVs) are released during the storage of red blood cell (RBC) concentrates and might play adverse or beneficial roles throughout the utilization of blood products (transfusion). Knowledge of EV release associated factors and mechanism amends blood product management. In the present work the impact of storage time and medium (blood preserving additive vs isotonic phosphate buffer) on the composition, size, and concentration of EVs was studied using attenuated total reflection infrared (ATR-IR) spectroscopy, microfluidic resistive pulse sensing (MRPS) and freeze-fraction combined transmission electron micrography (FF-TEM). The spectroscopic protein-to-lipid ratio based on amide and the C–H stretching band intensity ratio indicated the formation of various vesicle subpopulations depending on storage conditions. After short storage, nanoparticles with high relative protein content were detected. Spectral analysis also suggested differences in lipid and protein composition, too. The fingerprint region (from 1300 to 1000 cm^−1^) of the IR spectra furnishes additional information about the biomolecular composition of RBC-derived EVs (REVs) such as adenosine triphosphate (ATP), lactose, glucose, and oxidized hemoglobin. The difference between the vesicle subpopulations reveals the complexity of the REV formation mechanism. IR spectroscopy, as a quick, cost-effective, and label-free technique provides valuable novel biochemical insight and might be used complementary to traditional omics approaches on EVs.

## Introduction

Vesiculation is a normal route for red blood cells (erythrocytes, RBCs) to eliminate specific harmful components such as denatured hemoglobin, band 3 neoantigens or immunoglobulin G that tend to accumulate during their lifespan^1^. Human red blood cells typically lose 20% of their membrane area during their lifetime. Extracellular vesicles (microparticles or microvesicles) are generated during normal aging of RBCs by budding the plasma membrane due to complement-mediated calcium influx, followed by vesicle shedding. This phenomenon takes place both in vivo and in vitro; in the latter case red blood cell derived extracellular vesicles (REVs) accumulated during storage in blood products might have proinflammatory and procoagulant effects and may play a role in adverse transfusion events and posttransfusion outcomes^2–5^. Storage-induced REVs carry antigens and signaling molecules e.g. tissue factor, immunoglobulin G (IgG), phosphatidylserine (PS), hemoglobin, and heme, that in transfusion settings can affect immune responses through interactions with blood cells, endothelium and clearance systems^6^. Procoagulant activity of REVs formed during RBC storage was also extensively studied^4,7–9^. REVs isolated from RBC units on days 7, 14, 21, 28, 35 and 42 were quantified and characterized by high resolution flow cytometry. Thrombin generation was followed by clotting time and procoagulant activity assays^7,8,10^. While the number of REVs is increasing continuously, in most of the studies on day 0 and day 7 no difference in clotting time compared with PBS control was observed, but starting from day 14, the clotting time shortened continuously. Bouchard and coworkers used fluorescence labellings against both glycophorin A (RBC membrane marker) and phosphatidylserine (PS)^10^. They concluded that from day 7 to day 42 of storage, the amount of glycophorin A positive and PS positive REVs varied over time and the number of PS positive REVs did not correlate with thrombin formation. Recently, Almizraq and coworkers recommended the extracellular vesicle content as an additional product quality indicator in RBC concentrate screening^11^. Furthermore, they revealed that both the concentration and size-profile of EVs in stored red blood cell units are markedly influenced by blood manufacturing methods, storage time and the method of detection^12–14^.

The mechanism of storage-induced vesicle formation from red blood cells has been investigated intensively^1,15,16^. RBC-derived EVs are formed at cytoskeleton-free regions of the cell membrane and vesiculation is suggested to be a lipid raft based process induced by ATP loss (eryptosis model)^17,18^. This is supported by the fact that depletion of ATP resources leads to an increase in intracellular calcium levels and alters the activity of key plasma membrane proteins producing membrane lipid asymmetry. The latter results in turn in increased exposure of anionic phospholipids, particularly phosphatidylserine (PS), in the outer leaflet of the plasma membrane, so that subsequent vesiculation yields EVs exposing PS. In line with this, while reduced levels of lipid-raft proteins such as stomatin and flotillin were found in stored RBCs, stomatin was enriched in REVs^2,19^. Accordingly, incubation of RBCs with agents enhancing intracellular calcium levels such as Ca^2+^ ionophores (e.g. A23187), lysophosphatidic acid (LPA) or some phorbol esters (e.g. PMA) promoted EV release^20^, while incubation with a scramblase specific inhibitor (R5421), inhibiting PS externalization, significantly limited the release of EVs^21^.

Controversially, recent findings based on proteomics^22^ and phospholipidomics^23^ have suggested that the main mechanism of storage-induced RBC vesiculation is linked to the membrane protein band 3 rather than to rafts. Band 3, the major protein component of the RBC membrane, is considered to play key roles in membrane reorganization during RBC storage (band 3 clustering model)^5,22^. Indeed, band 3 and actin were detected in REVs but other integral membrane and cytoskeletal proteins, such as spectrin and ankyrin were not found^24^. Alterations in band 3 aggregation/degradation related to RBC aging were also revealed^25^. All these findings suggest that the breakdown of connections between the cytoskeleton and the lipid bilayer provided by band 3—ankyrin interaction might be a crucial step in the vesiculation process^1,19,22^. Prudent et al. reported that flotillin can associate with band 3 in stored RBCs, further confirming the involvement of band 3 complexes in microvesiculation^26^.

The interpretation of the large amount of new information obtained from omics data, however, might easily be bottlenecked by the high variability of RBCs collected from different donors stored under hypothermic conditions (1–6 °C)^27^. Therefore, in the present study we investigated systematically REVs isolated from RBC concentrates, stored in saline-adenine-glucose-mannitol (SAGM) additive solution under hypothermic conditions (4 °C)^28,29^. SAGM is currently the standard additive solution used in blood banking infrastructures in the EU. Glucose and adenine serve to maintain adenosine triphosphate (ATP) and 2,3-diphosphoglycerate (2,3-DPG) levels, while mannitol has functions in maintaining membrane integrity and combating hemolysis^30,31^. To monitor the effect of storage time, REV isolations were performed at different time points (at 3, 8 and 21 days of storage), and the isolates were characterized. For purposes of comparison, the same isolation and characterization procedure was carried out on REVs derived from RBCs stored in isotonic phosphate buffered saline (PBS) medium (pH = 7.4), a common buffer for biochemical and biophysical experiments^32–34^.

During RBC storage lesions, compositional changes due to the release of self-assembled nanostructures might lead to the formation of versatile novel morphologies. Here we performed a multilevel characterization of these morphologies by applying a combined approach utilizing unconventional biophysical techniques, such as attenuated total reflection infrared spectroscopy (ATR-IR), microfluidic resistive pulse sensing (MRPS), size exclusion chromatography coupled with on-line fluorescence detection (Flu-SEC) and freeze-fracture combined with transmission electron microscopy (FF-TEM). Special emphasis was placed on monitoring biochemical alterations by IR spectroscopy providing a nondestructive label-free investigation frame of extracellular vesicles. Although MS-based omics approach can provide more detailed speciation within a given class of biomolecules (proteins, lipids, metabolites), is time- and instrument-consuming and need complex sample preparation, that may alter the ratio of specimens. On contrary, IR spectroscopy is a relatively simple, rapid method associated with structural changes at the molecular level, which can provide information from proteins, lipids, organophosphates, carbohydrates from one experiment^35–38^.

## Results

**Figure 1:**
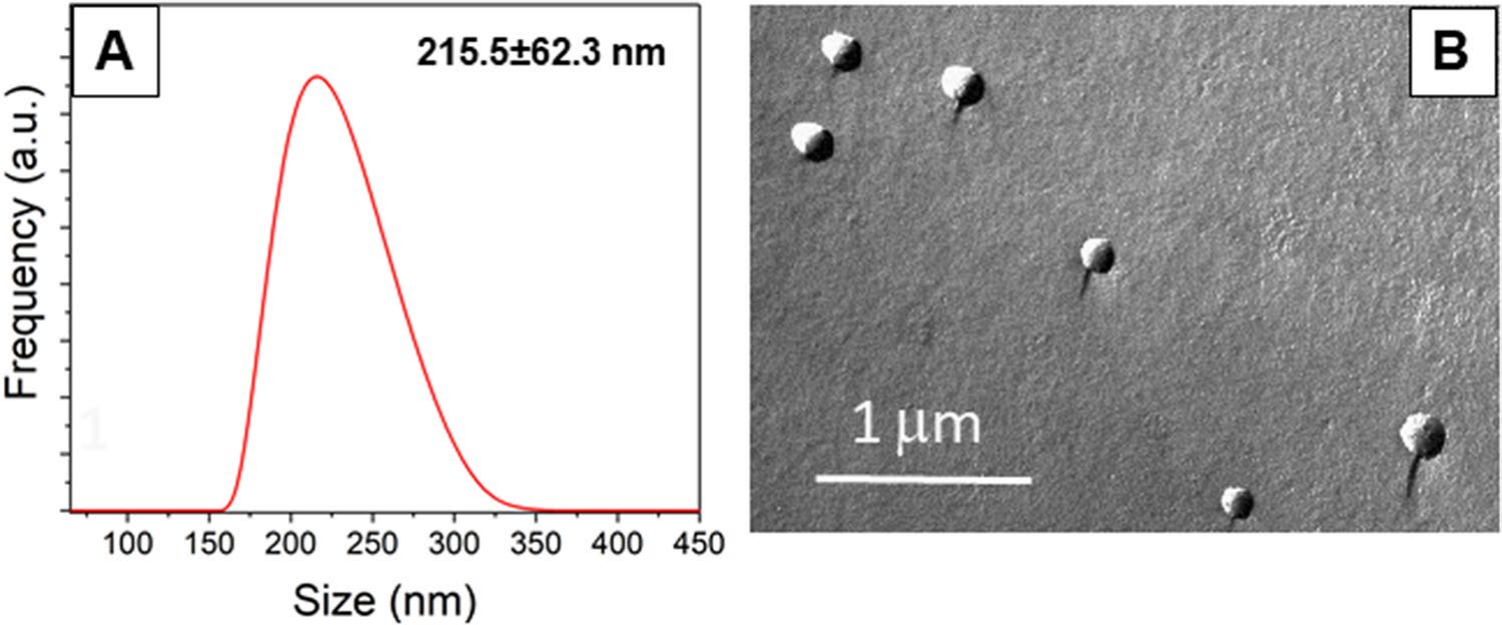
Characterization of REV samples isolated from stored (SAGM) RBCs after 21 days: (**A**) hydrodynamic diameter (with standard deviation) and size distribution measured by DLS; (**B**) a typical FF-TEM image.

The success of REV isolation was confirmed by dynamic light scattering (DLS) and freeze-fracture combined TEM (FF-TEM) images. In general, heterogeneously-sized particles were detected with an average diameter of ~ 200 nm (Fig. 1).

**Figure 2:**
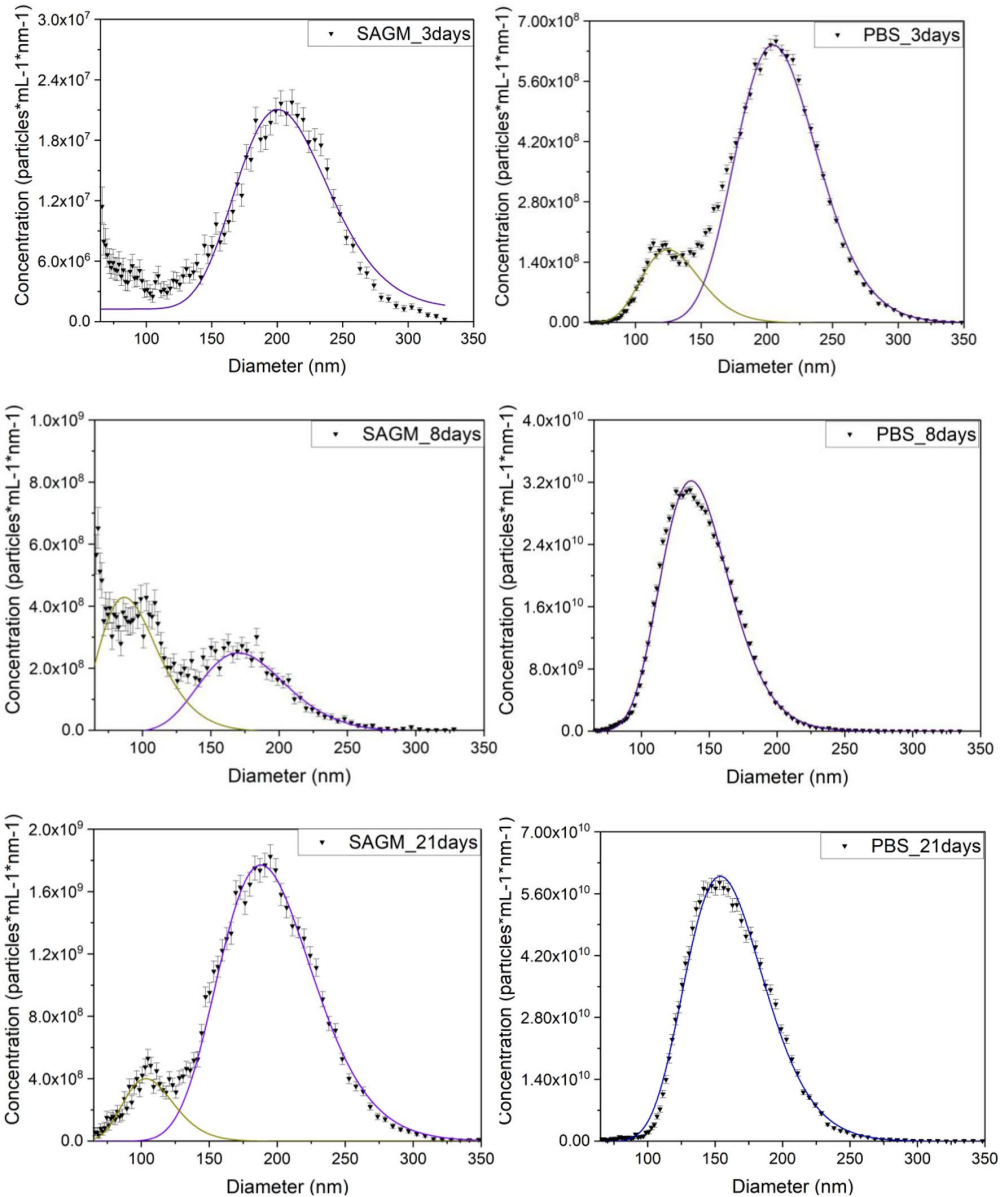
Size distribution and concentration of REV samples, measured by microfluidic resistive pulse sensing (MRPS). Error bars correspond to the counting error of each size bin.

Despite the simplicity and speed of typical DLS measurements, the inability of the method to reliably characterize polydisperse size distribution makes it less suitable for quantitative analysis of biofluid-originated particles^39^. Microfluidic resistive pulse sensing (MRPS) offers a quantitative size distribution determination based on the Coulter principle: it detects individual nanoparticles by measuring changes in electrical current as each particle passes through a nanopore^40,41^. Figure 2 shows the size distribution of REV samples obtained at different isolation times, fitted by log-normal distributions.

Based on the MRPS results (Fig. 2) a particle subpopulation with diameter of around 100 nm is also noticed. The satellite peaks on deconvoluted curves suggest that nanoparticles beside the REVs are also present. A bimodal particle distribution was also observed by Nguyen et al., investigating extracellular vesicles released from RBCs under conditions of stimulating Ca^2+^ uptake, using scanning electron microscopy (SEM) and atomic force microscopy (AFM). They identified two populations with around diameters of approximately 200 and 125 nm. The bimodal distribution is also witnessed in the case of REVs formed from RBCs in the early stage of storage (3 days) when these were stored in isotonic PBS buffer. In PBS buffer, however, after a one week or longer storage period, only almost monodisperse EVs with diameter of approximately 150 nm were detected.

**Figure 3:**
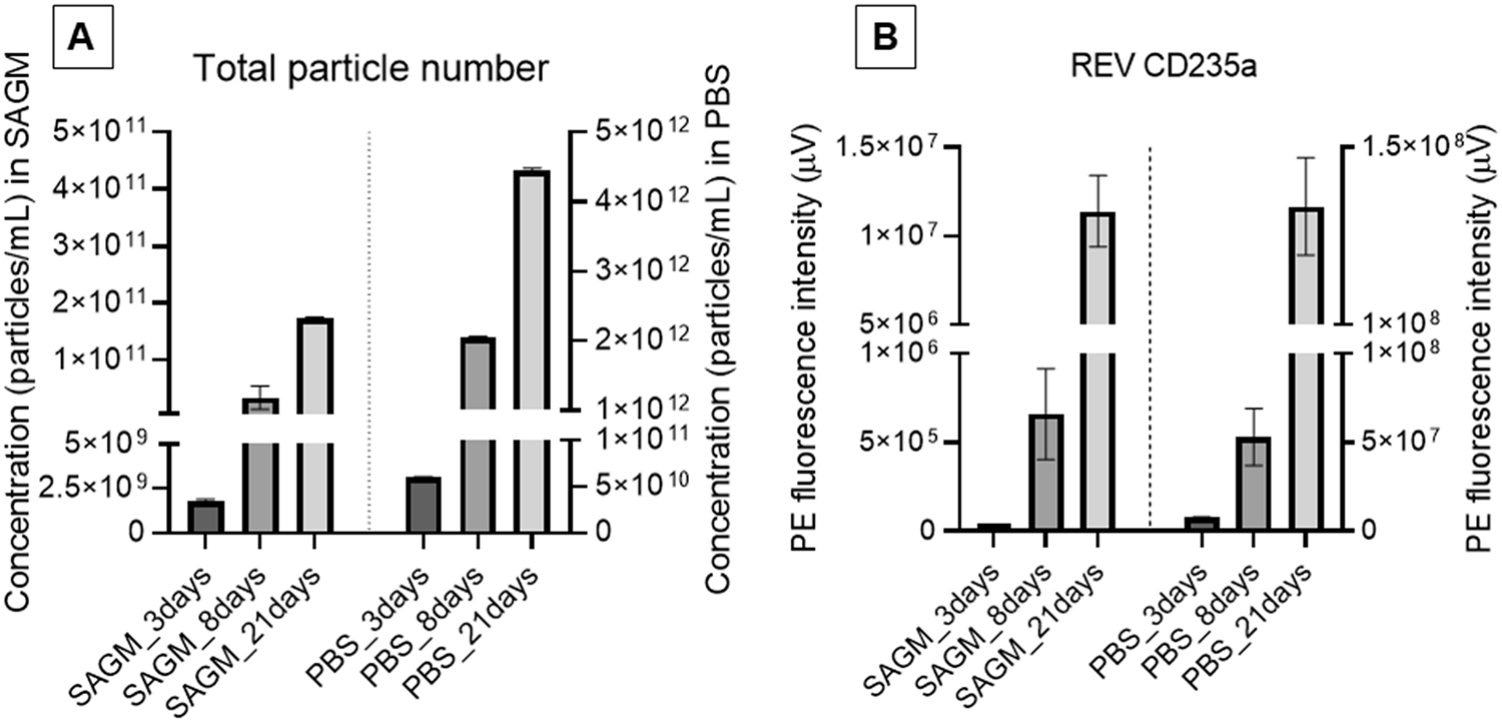
Total number of particles (nanoparticles and REVs) determined by MRPS measurements over the size range from 65 to 400 nm (**A**) and the peak height of the REV fluorescence peak using PE-CD235a (**B**). Error bars correspond to standard deviations.

With increasing storage time, the total particle concentration increases; the SAGM and PBS milieu caused the same tendency. Almizraq and coworkers, using flow cytometry and tunable resistive pulse sensing methods, also recognized an increase in total REV concentration with increasing hypothermic storage time^13^. Indeed, we observed a linear increment by orders of magnitude in total particle concentration with storage time (Fig. 3A). It is interesting to note, however, that in PBS the total particle (vesicle and nanoparticle) formation is more elevated: the number of total particles generated from RBCs stored in SAGM medium is more then tenfold less, than their counterparts from isotonic PBS buffer storage (1.77^.^10^9^ ± 6.12^.^10^7^ vs 6.03^.^10^10^ ± 4.46^.^10^8^, 4.41^.^10^10^ ± 2.53^.^10^9^ vs 2.05^.^10^12^ ± 1.53^.^10^10^ and 1.74^.^10^11^ ± 1.63^.^10^9^ vs 4.46^.^10^12^ ± 2.76^.^10^10^ for 3 days, 8 days and 21 days samples, respectively). Accordingly, RBCs stored in SAGM additive solution undergo less expressed vesiculation. This is in line with the explanation of Salzer et al.^2^, that storage induced microvesicles emerge from the loss of ATP-dependent translocase^42^. Indeed, the currently used saline-adenine-glucose-mannitol (SAGM) solution for blood banking, which contains substrates for 2,3-DFG and ATP production, preserves the integrity of RBCs for a longer time^43^.

The MRPS analysis was extended with size exclusion chromatography combined with fluorescence detection (Flu-SEC). REV samples were labeled with a fluorochrome conjugated antibody (PE-antiCD235a) against glycophorin A, a characteristic membrane protein of RBCs. Figure 3B shows the fluorescence intensity of phycoerythrin (PE-antiCD235a) bound to RBC derived vesicles. Based on the work of Kitka et al.^44^, the peak height can be directly correlated to the amount of REVs presuming identical antigen density. However, this assumption is not trivial for REVs isolated at different storage times and medium. Nevertehless, after the comparison of the results of MRPS and Flu-SEC we can conclude that the REV concentration increases with storage time, and it is higher when RBCs are stored in PBS instead of SAGM.

**Figure 4:**
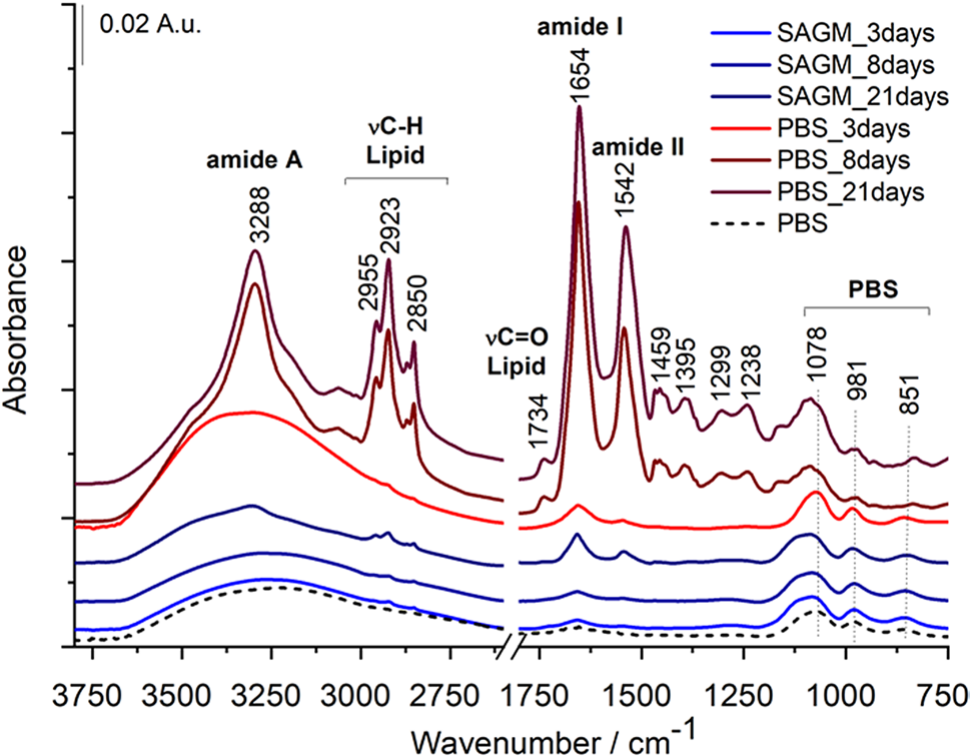
Representative raw IR spectra of purified REV samples. For better visualization spectra are shifted vertically. At early isolation time points the spectra are dominated by the PBS buffer background.

To get insight into the chemical composition of REV samples, IR spectra of different REV subpopulations were analyzed (Fig. 4). Bands corresponding to amide A, amide I and amide II vibrations (at 3288, 1654 and 1542 cm^−1^, respectively) of the protein backbone usually dominate the spectra^35,45^. In addition to the amide bands of protein components, characteristic bands of phospholipids are also present (Table 1). The sharp bands at 2923 and 2850 cm^−1^ correspond to the methylene antisymmetric and symmetric stretching vibrations of the long lipid acyl chains, respectively, while the band at 1734 cm^−1^ belongs to the ester carbonyl of phospholipids, triglycerides and cholesterol esters^35,44^. Smaller bands at 1459 and 1395 cm^−1^ corresponds to the bending vibrations of CH_2_ and CH_3_ groups from lipid chains. The bands at 1299 and 1238 cm^−1^ can be assigned to PO_2_^-^ stretching bands of phospholipids, however, the C–N stretching mode of proteins (amide III band) appears also in this spectral region. The lower frequency part is mostly masked by the buffer vibrations. Indeed, the triple bands at 1078, 981 and 851 cm^−1^ belong to the inorganic phosphate vibrations of PBS buffer. In some cases, REV spectrum analysis might enroll difficulties due to the low EV concentrations where the features of PBS are dominating the spectrum.Thus, careful buffer background subtraction is indispensable for detailed spectral analysis.

**Table 1:** Position and assignment of typical IR bands of REVs.

Wavenumber (cm^−1^)	Vibrational mode	Assigment
3288	N–H stretching (amide A) overlapped with –OH stretching	Proteins
2955	CH_3_ antisymmetric stretching	Lipids and proteins, carbohydrates
2923	CH_2_ antisymmetric	Lipids and side chains of proteins
2850	CH_2_ symmetric stretching	Lipids and side chains of proteins
1734	C=O stretching	Phospholipids, triglycerides, cholesterol esters
1654	C=O stretching (amide I)	Proteins
1542	N–H deformation with C–N stretching (amide II)	Proteins
1459	CH_2_ deformation	Lipids
1395	CH_3_ deformation	Lipids and side chains of proteins, carbohydrates
1299 1238	PO_2_^-^ antisymmetric stretching and C–N stretching (amide III)	Lipids and proteins

**Figure 5:**
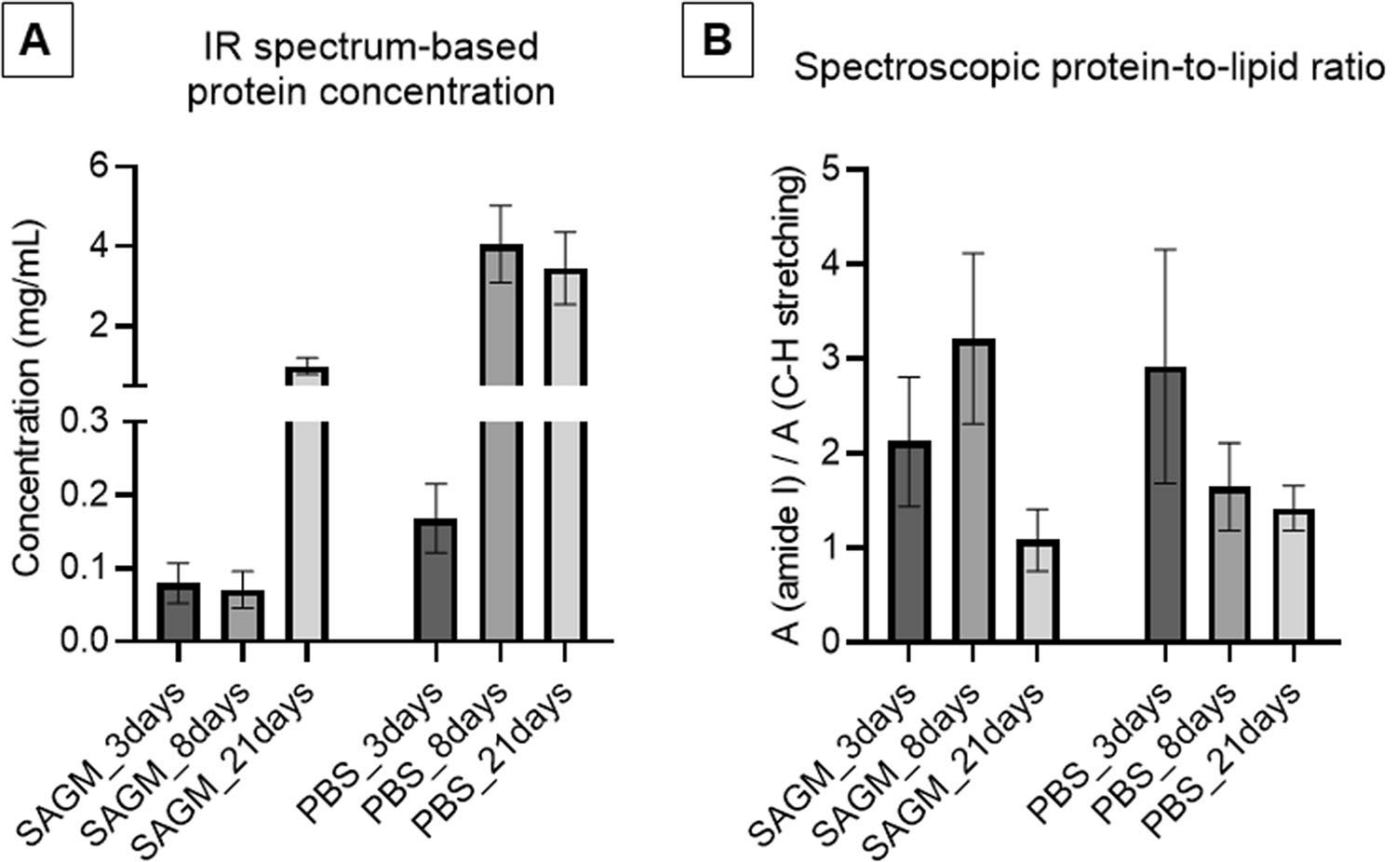
Total protein concentrations (**A**) and ‘spectroscopic protein-to-lipid ratio’ (**B**) calculated from the IR spectra of REV samples isolated at different time points. Error bars denote standard deviations.

Determination of the total protein concentration is one of the most straightforward ways to characterize EVs. Total protein content can be assessed by standard colorimetric protein assays (bicinchoninic acid (BCA) or Bradford assay). Quite recently, we introduced a label-free method to estimate the total protein concentration of intact EVs, using ATR-IR spectroscopy^46^. As the intense IR band of proteins (amide I band at approximately 1654 cm^−1^) is proportional to the number of peptide groups, using a proper calibration curve and careful spectrum evaluation protocol, the total protein content can be evaluated from the IR spectra of REV samples (Fig. 5A). The total protein concentration was further verified by common Bradford colorimetric assay resulting in very similar results as shown in Fig. S1.

Surprisingly, the protein concentration did not increase continuously with storage time. The different patterns obtained for protein content and particle number suggest that the vesicles (particles) from different subpopulations might also differ regarding their chemical composition, too (Fig. S2).

Further advantage of IR spectroscopy is to provide absorption bands of protein and lipid components simultaneously. As they are well distinguishable, a spectroscopic protein-to-lipid ratio was suggested as a parameter for EV characterization^35,47^. The ‘spectroscopic protein-to-lipid’ ratio values for the different REV samples, calculated as the ratio of integrated areas of the amide I band and that of the C–H stretching (from 3040 to 2800 cm^−1^ wavenumber region), are presented in Fig. 5B. We must stress, that as long as the protein concentration is in absolute value (mg/mL), the ‘spectroscopic protein-to-lipid ratio’ is an index number for EV characterization.

**Figure 6:**
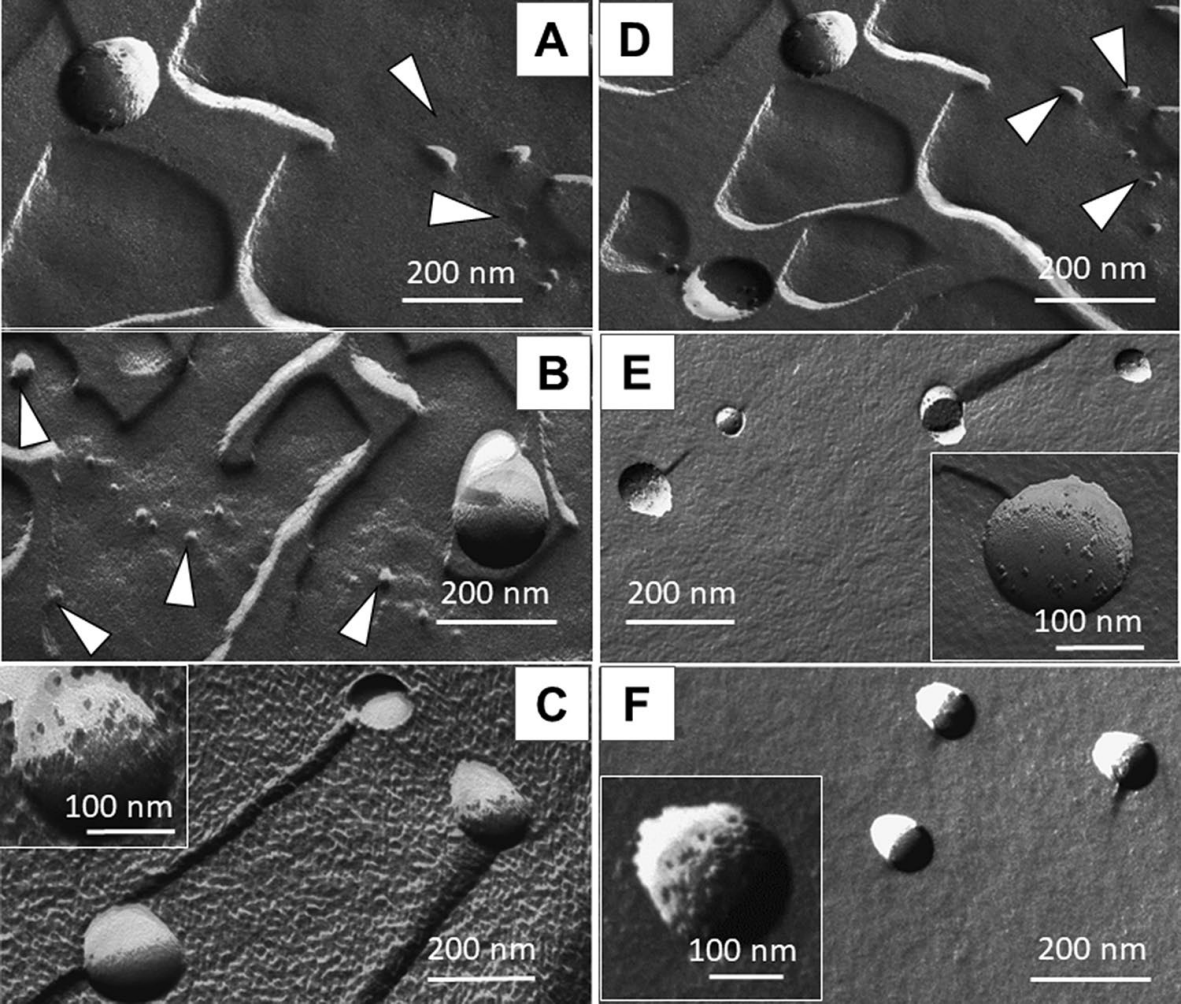
Electron micrographs obtained by freeze-fracture combined with TEM of REV samples isolated from RBCs stored in SAGM at 3-days (**A**), 8-days (**B**) and 21-days (**C**), and in PBS at 3-days (**D**), 8-days (**E**) and 21-days (**F**). Arrows emphasize small particles, while insets show selected images of single vesicles.

The majority of REV samples provided spectroscopic P/L values in the range from 0.6 to 2, which is typical for RBC derived EV samples based on our previous results^35,44^. Outlier data (SAGM_3days, SAGM_8days and PBS_3days) suggest that at short storage times, a relatively small amount of nanoparticles, rich in proteins, are formed. This is in perfect line with the presence of small nanoparticles, witnessed in FF-TEM images (Fig. 6).

Despite that observed in a limited number on the micrographs (Fig. 6**),** typical REVs with almost regular spherical shapes having 200 nm average diameters are witnessed. Their surface is randomly covered by smaller particles 5–15 nm in diameter, presumable membrane proteins or protein aggregates^40^. This peculiar surface pattern is more pronounced in the case of storage in PBS solution. On the images corresponding to the REV sample isolated at early (3-days) time points, several smaller formations with sizes ranging between 20–80 nm can be discovered. The presence of smaller vesicles (with an average diameter of approximately 80 nm), called ‘nanovesicles’ was also mentioned by Bosman et al.^24^ after ultracentrifugation (100,000 × *g*) of the supernatant of stored RBC concentrates. Performing detailed LC–MS/MS analysis and proteomics data evaluation the authors revealed differences in composition for microvesicles and nanovesicles; they concluded that the two populations might be generated by different processes. Based on the experienced high protein-to-lipid ratio and compared with our previous results on spectroscopic P/L values on small vesicles (exosomes)^35^, we speculate that the observed small particles might be protein complexes rather than vesicles. By increasing the storage time (21-days) before isolation, the nanoparticles were no longer detected (Fig. 6C,F). It seems plausible that the release of smaller entities is characteristic of early storage lesion of RBCs and with an increasing number of larger vesicles, these nanoparticles fuse with, or connect to vesicles, as indicated by the spectroscopic protein-to-lipid ratio typical for EVs. Notably, in PBS these nanoparticles were not detected after 8 days of storage. These findings are in fairly good accordance with MRPS results and might serve as an explanation for elevated spectroscopic protein-to-lipid ratios.

As the position and shape of IR absorption bands depend on intra- and intermolecular interactions, the IR spectroscopy method can be sensitive to minor biomolecular changes. Detailed spectral analysis was performed using the second derivatives of the spectra of REV samples. We focused on three spectral regions, related to C–H stretching vibrations (from 3050 to 2800 cm^−1^), to amide I and amide II bands of protein backbones (from 1700 to 1500 cm^−1^) and the fingerprint region (from 1300 to 1000 cm^−1^) containing phosphate and ether/ester vibrations.

**Figure 7:**
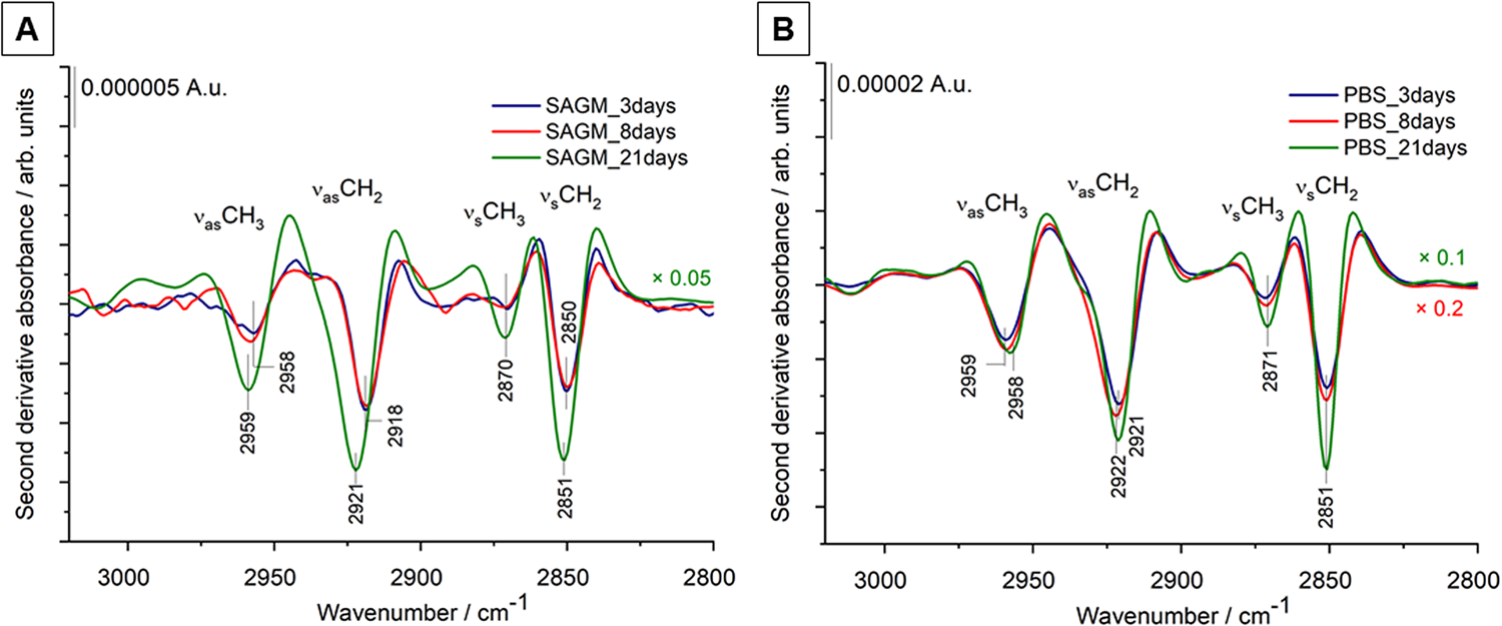
Second derivatives of average IR spectra (from 3050 to 2800 cm^−1^) of REV samples isolated from RBCs stored in SAGM and PBS medium. At least 3 spectra were averaged corresponding to 3 different biological replicas (starting from different donors). For better visualization, the spectra of the SAGM_21days, PBS_8days and PBS_21 days samples are multiplied by 0.05, 0.2 and 0.1, respectively.

The antisymmetric and symmetric stretching of the long acyl chains of lipids (ν_as_CH_2_ and ν_s_CH_2_ around 2920 cm^−1^ and 2850 cm^−1^, respectively) are sensitive parameters of lipid ordering and packing, forming the lipid bilayer^48–51^. As revealed by the second derivatives in Fig. 7A, there are slight but definite differences in the positions of the CH_2_ stretching vibration bands regarding REVs from early storage. This further confirms the previous findings suggesting that in majority particles with slightly different lipid composition and/or organization are formed in the first 8 days using SAGM additives. In PBS buffer, no significant difference regarding lipid peak positions was observed, in line with the elevated vesicle level even at the early isolation point. It is worth to note, that after 21 storage days, the C–H stretching spectral features are similar, irrespective of the storage medium.

**Figure 8:**
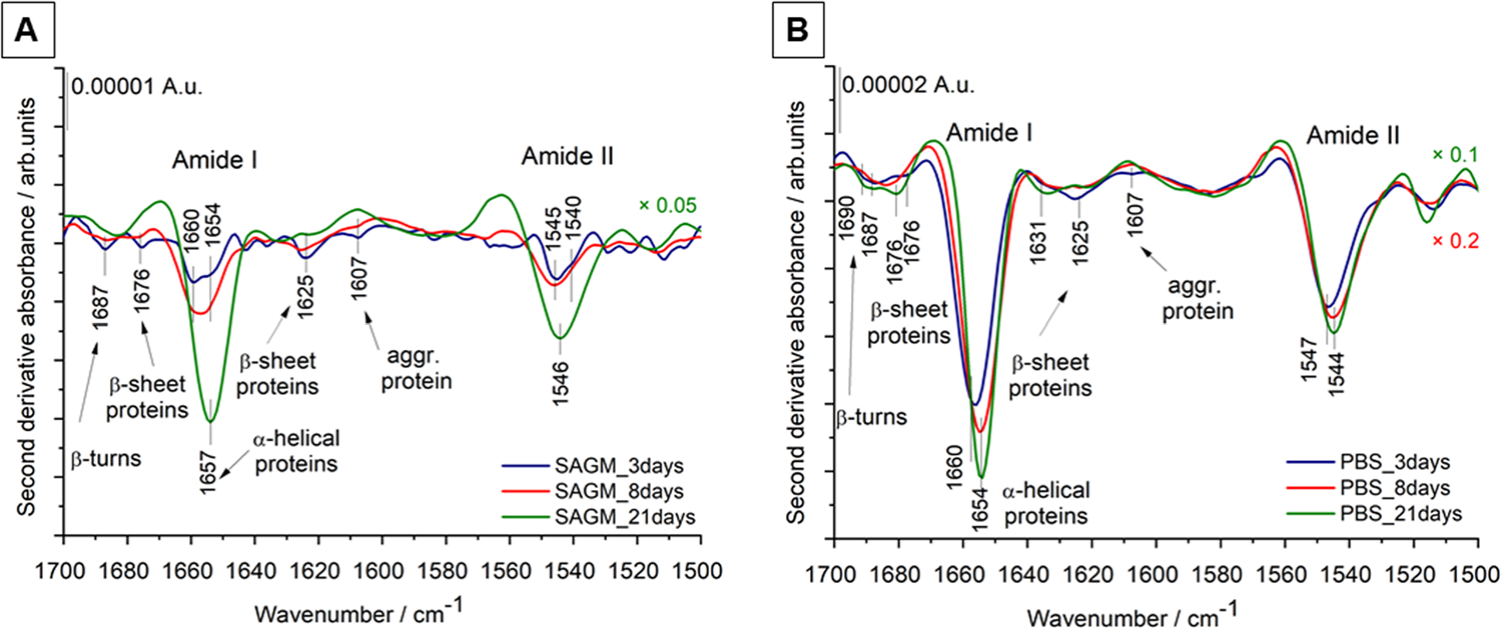
Second derivatives of average IR spectra (from 1700 to 1500 cm^−1^) of REV samples isolated from RBCs stored in SAGM and PBS medium. At least 3 spectra were averaged corresponding to 3 different biological replicas (starting from different donors). For better visualization, the spectra of the SAGM_21days, PBS_8days and PBS_21 days samples are multiplied by 0.05, 0.2 and 0.1, respectively.

The spectral region from 1700 to 1500 cm^−1^ is characteristic to protein components, comprising the two prominent amide absorptions, termed amide I and amide II. Amide I originates mainly from the C=O stretching of the peptide backbone and is used to assess the secondary structure of the proteins. The envelope of amide I can be resolved to individual band components (by using second derivative IR spectra, Fourier self-deconvolution, e.g.) which are characteristic to the α-helical, β-sheet, turn, random, etc. content of the proteins. Figure 8 highlights the spectral differences of REV samples. Early storage samples exhibit a versatility of protein secondary structures; derivative IR spectra of SAGM-stored REV samples show a variability of helical structures pointed by a double peak at 1660 and 1654 cm^−1^. These changes are also reflected in the amide II band region, arising from N–H bending vibrations and C–N vibrations of the peptide groups (Fig. 7A). In addition to the helical conformations, bands belonging to β-sheets (at 1625 and 1676 cm^−1^) and β-turns (at 1687 cm^−1^) are also witnessed. The derivative minimum at 1607 cm^−1^ can be assigned to nonnative intermolecular β-sheets, suggesting the presence of aggregated proteins^35,52^. REVs isolated after 21-days of storage (SAGM_21days), are characterized by a dominating helical contribution (amide I band component centered at 1657 cm^−1^). Likewise, for REVs formed in PBS, the helical protein structure is prevalent. This is consistent with the increased relative hemoglobin content (predominantly α-helix protein) of PBS-stored REVs (Fig. S3). At the early isolation point (PBS_3days), however, the presence of derivative peaks corresponding to β-sheets, β-turns and aggregated proteins supports the presence of protein-rich particles, similar to REVs from SAGM additive solution. REVs isolated after 21 days of storage show very similar spectral features, suggesting that the protein composition of the released REVs tends to be comparable.

**Figure 9:**
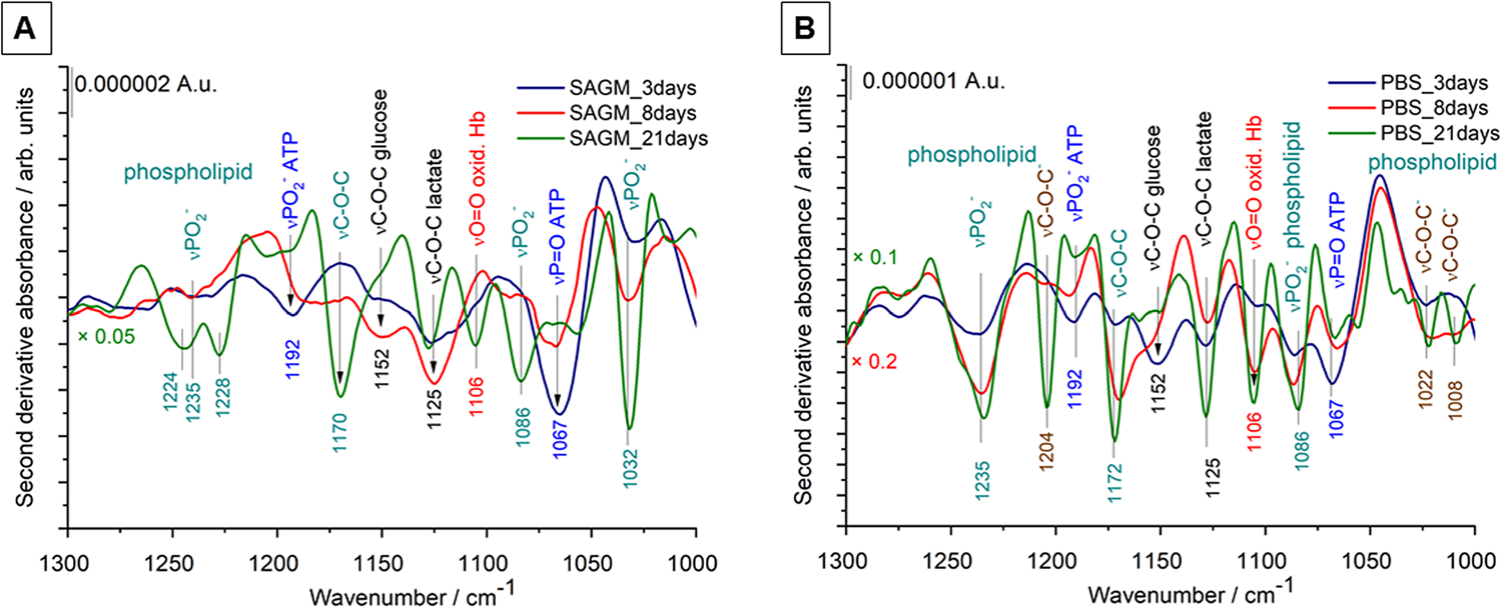
Second derivatives of average IR spectra (from 1300 to 1000 cm^−1^) of REV samples isolated from RBCs stored in SAGM and PBS medium. At least 3 spectra were averaged corresponding to 3 different biological replicas (starting from different donors). For better visualization, the spectra of the SAGM_21days, PBS_8days and PBS_21 days samples are multiplied by 0.05, 0.1 and 0.2, respectively.

The wavenumber region below 1300 cm^−1^ of an IR spectrum is commonly called the fingerprint region and usually contains a complicated series of various absorption bands unique for a particular sample. The fingerprint spectral region of REV samples ranging from 1300 to 1000 cm^−1^ is presented as second derivatives in Fig. 9. This region may contain the P–O and C–O stretching vibrations of phospholipids, triglycerides, cholesterol ester and sugars, such as glucose, and lactate, all potentially present in blood related samples. We must mention, however, that inorganic phosphate can have a great contribution in this region, so correction for the PBS buffer components is crucial.

The fingerprint region of the early isolated REV sample (SAGM_3days) differs from that of SAGM-8 days and SAGM-21 days (Fig. 9A). The second derivative spectra are dominated by peaks at 1192 and 1067 cm^−1^, assigned to antisymmetric PO_2_^−^ and P=O stretching, respectively, of polyphosphate in adenosine triphosphate^53,54^. The spectral features of ATP can also be witnessed for early REV samples isolated from PBS buffer (Fig. 9B). REV samples isolated from PBS-stored RBCs with higher amounts of vesicles have more intense phosphate bands around 1235 and 1080 cm^−1^, corresponding to antisymmetric and symmetric PO_2_ stretching vibrations, respectively, of lipid phosphate esters. The broader bands around 1020–1030 cm^−1^ might correspond to the phosphate diester groups of the phospholipid headgroup moiety. The band at 1170 cm^−1^, also more pronounced in REVs obtained from PBS storage, can be assigned to the C–O–C stretching of phospholipids, cholesterol esters and/or triglycerides. The higher intensity of the phospholipid-related bands in this wavenumber region is in line with the more expressed ethylene stretching vibrations of the corresponding acyl chain moieties (Fig. 7).

Well-defined bands at 1152 cm^−1^ and 1125 cm^−1^ can be identified in the second derivative spectrum of REV samples isolated after 3-days of storage. According to Pistorius et al.^54^ we assigned the band at 1152 cm^−1^ to the C–O–C stretching of glucose; they selected the same band to characterize the glucose level of RBC concentrates using IR spectroscopy. Indeed, the latest proteomics results on REV samples performed by Bosman and coworkers^22^ corroborate the presence of proteins related to the energy metabolism of RBCs. It is interesting to note, that for the rest of the REVs the glucose-related band is suppressed and the band at 1125 cm^−1^, corresponding to lactate is increasing^54^. The relative intensity of the lactate band was the highest for the 8-days SAGM-stored samples. As the REV metabolome reflects the kinetics of RBC energy metabolism, it seems plausible, that a connection exists between the long-drawn glycolysis pathway and delayed vesiculation. Moreover, at short storage times, REV samples might also contain adenosine triphosphate (ATP), witnessed by the presence of bands at 1192 and 1067 cm^−1^, belonging to PO_2_^−^ and P=O of polyphosphates^54^.

Another marker IR band is the one at 1106 cm^−1^. This band relates to oxidized hemoglobin^55^ and is assigned to the nonsymmetrical bonding between heme iron and oxygen^56^. The increased intensity of the oxyhemoglobin band in the spectra of PBS-stored REVs is in line with the results of UV–Vis spectroscopy indicating an increased relative hemoglobin concentration (Fig. S3).

The maximum allowed storage duration of RBC products is usually 42 days^57^. However, to decrease general transfusion-related risks, based on special transfusion requirements a classification of RBCs as ^“^young” (< 14–21 days) *vs* “old” (> 21 days) is in use^58^. REVs isolated from RBCs stored in SAGM for 21-days mimic blood banking conditions. It is worth to note that spectral changes due to different storage media are not as significant as those for early isolated REVs. Only small, but notable changes are observed in the fingerprint spectral region of REVs (Fig. 8). Bands in the 1245–1220 cm^−1^ wavenumber region and at 1032 cm^−1^ correspond to antisymmetric PO_2_^-^ and lipid ester (R–O–P–O–R’) stretching vibrations of phospholipids, respectively, and spectral variations suggest some differences in their lipid headgroup composition and/or geometry for the two REV samples. In the derivative spectrum of PBS-stored late REVs extra bands at 1204, 1022 and 1008 cm^−1^ can be observed. These bands might belong to carbohydrates/glycogens^54^. It seems plausible that during storage the vesicles are enriched not only in hemoglobin but also in red blood cell membrane specific glycoproteins such as band 3 or glycophorin A-D. Recent proteomics on REVs isolated at different storage times from blood banks shows that the enrichment of band 3 protein in the vesicles achieved a maximum after 3 weeks of storage^22^. Regarding our experiment we speculate that in PBS-stored REVs hemoglobin and band 3 might also be present at a higher percentage than in SAGM-related REVs. Further bands in the derivative spectra can be assigned to the C–O–C of cholesterol esters/phospholipids/triglycerides (around 1170 cm^−1^), to lactate (1125 cm^−1^) and oxyhemoglobin (1106 cm^−1^). The hemoglobin content of REV samples also varies as a function of the storage conditions. In general, the REVs from RBCs stored in isotonic PBS buffer solution contained higher levels of hemoglobin and its relative amount increases with storage time, reflected also by the growth of the oxyhemoglobin band at 1106 cm^−1^.

## Conclusion

Detailed IR spectroscopic investigation of red blood cell derived extracellular vesicles (REVs) revealed a series of traceable spectral changes depending on storage time and medium.

Short storage of RBCs leads to a versatility of particles: in addition to extracellular vesicles (microparticles) small (nano)particles are also released resulting in a higher spectroscopic protein-to-lipid ratio. In these peculiar cases, the increased relative protein concentration, associated with the presence of protein-rich small particles, was further corroborated by morphology- and size-related information provided by microfluidic resistive pulse sensing and electron microscopy. Extending RBC storage duration, the vesicle concentration, and their uniformity increase; the growing total protein concentration assessed from IR spectra meets the number of particles and the spectroscopic protein-to-lipid ratio indicates the presence of ‘pure’ extracellular vesicles. All these observations support the idea that early stage vesiculation/lesions might be driven by different mechanisms.

Samples from RBCs stored for 3-days contain nucleoside triphosphates, as indicated by the PO_2_^−^ and P=O bands at 1192 and 1067 cm^−1^ assigned from the second derivative IR spectra, respectively. These bands disappear from the spectra of REV samples isolated at later time points. Other IR markers, related to RBC metabolism, are the bands at 1152 and 1125 cm^−1^ in the fingerprint region, assigned to glucose and lactate, respectively. Glucose can be observed only for early REVs while the concentration of lactate shows an increasing trend with RBC storage time. Because RBCs lack mitochondria, they cannot use oxidative phosphorylation for ATP regeneration but have to rely on metabolizing glucose to lactate for energy supply^43^.

The storage medium also influences the vesicle characteristics. RBCs stored in phosphate-buffered saline (PBS, pH = 7.4) suffer more pronounced vesiculation as part of the storage lesion. IR spectroscopy is sensitive to compositional differences induced by alteration of the storage medium. REVs from PBS-stored RBCs have more hemoglobin reflected also in the IR spectra: the complex amide I band, characteristic for secondary structure exhibits dominating amount of α-helical proteins. The fingerprint region shows an IR marker at 1006 cm^−1^ of oxidized hemoglobin. The high vesicle concentration and uniformity of PBS-stored REVs, however, obtained even after 8 days of storage, create a suitable EV reference platform for further physico-chemical investigations and developments.

Here we demonstrate that IR analysis is a suitable tool to characterize EV samples and can provide new biophysical and biochemical insight in a nondestructive and reagent-free manner. Specific IR markers (e.g. protein-to-lipid ratio, relative intensity of the amide I band) can be correlated to the number and morphology of particles, while the position and intensity of selected vibrational bands can be informative for chemical composition. Although IR spectroscopy cannot compete with the detailed semiquantitative specifications provided by proteomics or lipidomics, due to its simplicity and minimal sample manipulation requirements, it can be complementary to traditional omics approaches.

## Experimental

### Preparation of red blood cell concentrates

Fresh anticoagulated blood was collected from healthy adult volunteers with informed consent using 6 ml K_3_EDTA tubes (Vacuette, Greiner Bio-One, Austria). The use of human blood samples was approved by the Scientific Ethics Committee of the Hungarian Health Scientific Council (ETT TUKEB 6449-2/2015) and during all procedures the guidelines and regulations of the Helsinki Declaration from 1975 were followed. 18 ml blood was collected from each donor and was used without pooling. Red blood cells (RBCs) were sedimented by centrifugation at 2500 × *g* for 10 min at 4 °C (Nüve NF 800R, swing out rotor). After removal of the plasma and the white blood cell containing buffy coat, the RBC pellet was washed 4 times with physiological NaCl solution. After the last sedimentation the RBC pellet was suspended in SAGM (saline-adenine-glucose-mannitol) additive^59^ or in isotonic PBS (pH = 7.4), apportioned in 6 aliquots (3 ml buffer to 1 ml RBC concentrate each aliquot) and kept under hypothermic conditions (at 4 °C).

### REVs isolation

RBC concentrates were used for EV isolation after storage for 3, 8 and 21 days. After two consecutive sedimentations (Nüve NF800R centrifuge, 2500 × *g*, 10 min and 3000 × *g*, 30 min, respectively) the supernatants were submitted to a 16,000 × *g* (Eppendorf 5415R, F45-24-11 rotor) centrifugation for 30 min. The pellets were washed with isotonic PBS buffer and the final pellet was suspended in 100 μl PBS. To purify the REVs from proteins or protein aggregates cosedimented, size exclusion chromatography (SEC) was performed by means of qEVsingle (IZON Science Ltd) columns (Fig. 1). According to the manufacturer’s protocol 100 μl EV suspension was loaded on the column, followed by elution with PBS. 200 μl fractions were collected. The first 5 fractions (1000 μl) corresponding to the void volume were discarded and the EVs were collected from fractions 6, 7 and 8 (verified also by dynamic light scattering measurements). The EV containing fractions were pooled and concentrated by centrifugation (16,000 × *g*, 30 min, Eppendorf 5415R, F45-24-11 rotor). The final pellet of purified REVs was resuspended in 200 μl PBS.

### Dynamic light scattering (DLS)

The presence of REVs during isolation and purification steps was justified by DLS measurements using a W130i dynamic light scattering apparatus (AvidNano, UK). 80 μl samples were used in a low-volume cuvette. Data analysis was performed using i-Size 3.0 software.

### Microfluidic resistive pulse sensing (MRPS)

Counting and sizing of REVs was accomplished with the microfluidic resistive pulse sensing technique using a Spectradyne nCS1 instrument (Spectradyne LLC, USA). Resistive pulse sensing is based on monitoring transient changes in electric current, also known as the Coulter principle, caused by particles (vesicles) passing through a narrow orifice. MRPS measurements were carried out using factory calibrated TS-400 microfluidic cartridges with a measurement range from 65 to 400 nm. REV samples were diluted with filtered (Amicon Ultra 0.5 mL MWCO100 kDa membrane filter, Merck Millipore, Germany) bovine serum albumin (BSA, Sigma-Aldrich, Hungary) solution in PBS buffer (pH = 7.4, Sigma-Aldrich, Hungary)^40,60^. The addition of BSA solution was adjusted to the REV concentration as follows: REV_3days:BSA (10 mg/mL) = 9:1 volume ratio; REV_8days:BSA (1 mg/mL) = 1:9 volume ratio and REV_21days:BSA (1 mg/mL) 1:9 volume ratio. A 100-fold dilution was necessary for the REV sample stored in PBS for 21 days.

### Attenuated total reflection infrared spectroscopy (ATR-IR)

ATR-IR spectra were collected using a Varian 2000 FTIR Scimitar Series spectrometer (Varian Inc, USA) equipped with a liquid nitrogen cooled mercury-cadmium-telluride (MCT) detector and with a ‘Golden Gate’ single reflection diamond ATR accessory (Specac Ltd, UK). A total of 3 μl of sample was mounted on top of the diamond ATR crystal and a thin dry film was obtained by slow evaporation of the buffer solvent under ambient conditions^61^. Spectra were collected at room temperature, immediately after drying the sample (within approximately 5 min). 64 scans were coadded at a nominal resolution of 2 cm^−1^. After each data acquisition ATR correction was performed. For all spectral manipulations the GRAMS/32 software package (Galactic Inc, USA) was used. Before detailed analysis the spectrum of PBS buffer was very carefully subtracted using the ‘de-wiggle’ algorithm by iteratively minimizing the first derivative of the subtraction results built in the GRAMS/32 software. Second derivatives for detailed analysis were obtained using the Savitzky-Golay algorithm with a 13-point smoothing window.

A spectroscopic protein-to-lipid ratio was calculated for all REV samples based on the integrated area of the amide I band (characteristic for protein content) and that of the C-H stretching region (from 3020 cm^−1^ to 2800 cm^−1^, typical mainly for phospholipid content). The provided spectroscopic P/L proved to be discriminant for different subtypes of EVs (small vs. medium or large EVs)^35^ and can also be used for assessment of REV sample purity^34,45,62^. As the amide I area is proportional to the number of peptide bonds in the proteins, the IR spectrum can be used to assess the total protein content of the REV samples, too^46^.

### Freeze-fracture transmission electron microscopy (FF-TEM)

FF-TEM allows the study of the morphology of EVs in their native environment, by rapid freezing of the sample and without any fixation or staining. Vesicle samples were mixed with glycerol (sample: glycerol at a 3:1 volume ratio) to avoid freezing artifacts. Approximately 1 μl of sample was pipetted onto a golden sample holder and suddenly frozen in liquid freon, then stored in liquid nitrogen. Fracturing was performed at − 100 °C in a Balzers freeze-fracture device (Balzers BAF 400D, Balzers AG, Liechtenstein). Replicas from the fractured surfaces were made by carbon-platinum shadowing, then washed with surfactant solution and distilled water. The replicas were placed on a 200 mesh copper grid and examined in a MORGAGNI 268D (FEI, The Netherlands) transmission electron microscope.

### UV–Vis spectroscopy

The hemoglobin content of REV samples originating from RBCs under different storage conditions was determined spectrophotometrically, measuring the absorbance at 414 nm in a quartz cuvette with a 1 cm optical path length. A Hewlett-Packard 8453 diode array UV–Vis spectrophotometer, equipped with Grant LTD 6G circulating water bath was used to ensure a constant temperature of 25 °C during measurements. Lyophilized hemoglobin (Sigma-Aldrich, Hungary) was used for the calibration curve.

### Size exclusion chromatography with on-line fluorescence detection (Flu-SEC)

The combined use of fluorescence detection and size exclusion chromatography (SEC) proved to be an orthogonal method for the quantification and phenotyping of EVs^34^. REV samples were labeled with a fluorochrome conjugated antibody (PE-antiCD235a) against glycophorin A, a characteristic membrane protein of RBCs. REV (20 μL) was incubated with 0.2 μL PE-antiCD235a (0.2 mg/mL, BioLegend, USA) for 30 min at 37 °C. 10 μL of labeled REV sample was injected into a Jasco HPLC system (Jasco Inc, Japan) using a PU-2089 pump and an FP-2020 fluorescence detector. Tricorn 5/200 glass columns (GE Healthcare Bio-Sciences AB, Sweden) filled with Sepharose CL-2B were used; the flow rate of the PBS eluent was 0.5 mL/min. Fluorescence chromatograms were collected at excitation and emission wavelengths (546/578 nm) corresponding to the phycoerythrin (PE) fluorochrome.

### Ethical approval and consent to participate

The use of human blood samples was approved by the Scientific and Research Ethics Committee of the Hungarian Medical Research Council (ETT TUKEB 6449-2/2015). EDTA-anticoagulated blood was collected from healthy volunteers with informed consent. During the entire investigation, we followed the guidelines and regulations of the Helsinki Declaration in 1975.

### Consent for publication

All donors and authors approved the manuscript to be published.

## Supplementary Information

Supplementary Information.
