# Supplementary material for: Storage conditions determine the characteristics of red blood cell derived extracellular vesicles
Source: Sci Rep. 2022 Jan 19;12:977. doi: 10.1038/s41598-022-04915-7 (PMC8770621; doi:10.1038/s41598-022-04915-7)
Supplement: Supplementary file 1 — Supplementary Information. [file 41598_2022_4915_MOESM1_ESM.docx]

**Supplementary Material**

**Storage conditions determine the characteristics of red blood cell derived extracellular vesicles**

Tímea Bebesi^1,2^, Diána Kitka^1,2^, Anikó Gaál^1^, Imola Csilla Szigyártó^1^, Róbert Deák^1^, Tamás Beke-Somfai^1^, Kitti Koprivanacz^3^, Tünde Juhász^1^, Attila Bóta^1^, Zoltán Varga^1*^, Judith Mihály^1,*^

*^1^Institute of Materials and Environmental Chemistry, Research Centre for Natural Sciences (RCNS), H-1117 Magyar tudósok körútja 2, Budapest, Hungary*

*^2^Hevesy György PhD School of Chemistry, ELTE Eötvös Loránd University,*

*H-1117 Pázmány Péter sétány 1/A, Budapest, Hungary*

*^3^Institute of Enzymology, Research Centre for Natural Sciences (RCNS), H-1117 Magyar tudósok körútja 2, Budapest, Hungary*

^*^Correspondence to: Judith Mihály, [*mihaly.judith@ttk.hu*](mailto:mihaly.judith@ttk.hu)*;* Varga Zoltán, [*varga.zoltan@ttk.hu*](mailto:varga.zoltan@ttk.hu)

**Fig.S1** Total protein content of REV samples determined by Bradford colorimetric assay

**Fig.S2** Protein-to-particle ratio obtained from total protein concentration and number of total particles. Results revealed that REVs isolated at different storage time and medium have different chemical composition; early formed particles from RBC stored in SAGM yielded the higher amount of protein

**Fig.S3** Relative hemoglobin content (hemoglobin concentration to total protein concentration ratio) in different REV samples
